# Supplementary figures and images for: Exploring pain interference with motor skill learning in humans: A systematic review
Source: PLoS One. 2022 Sep 13;17(9):e0274403. doi: 10.1371/journal.pone.0274403 (PMC9470002; doi:10.1371/journal.pone.0274403)

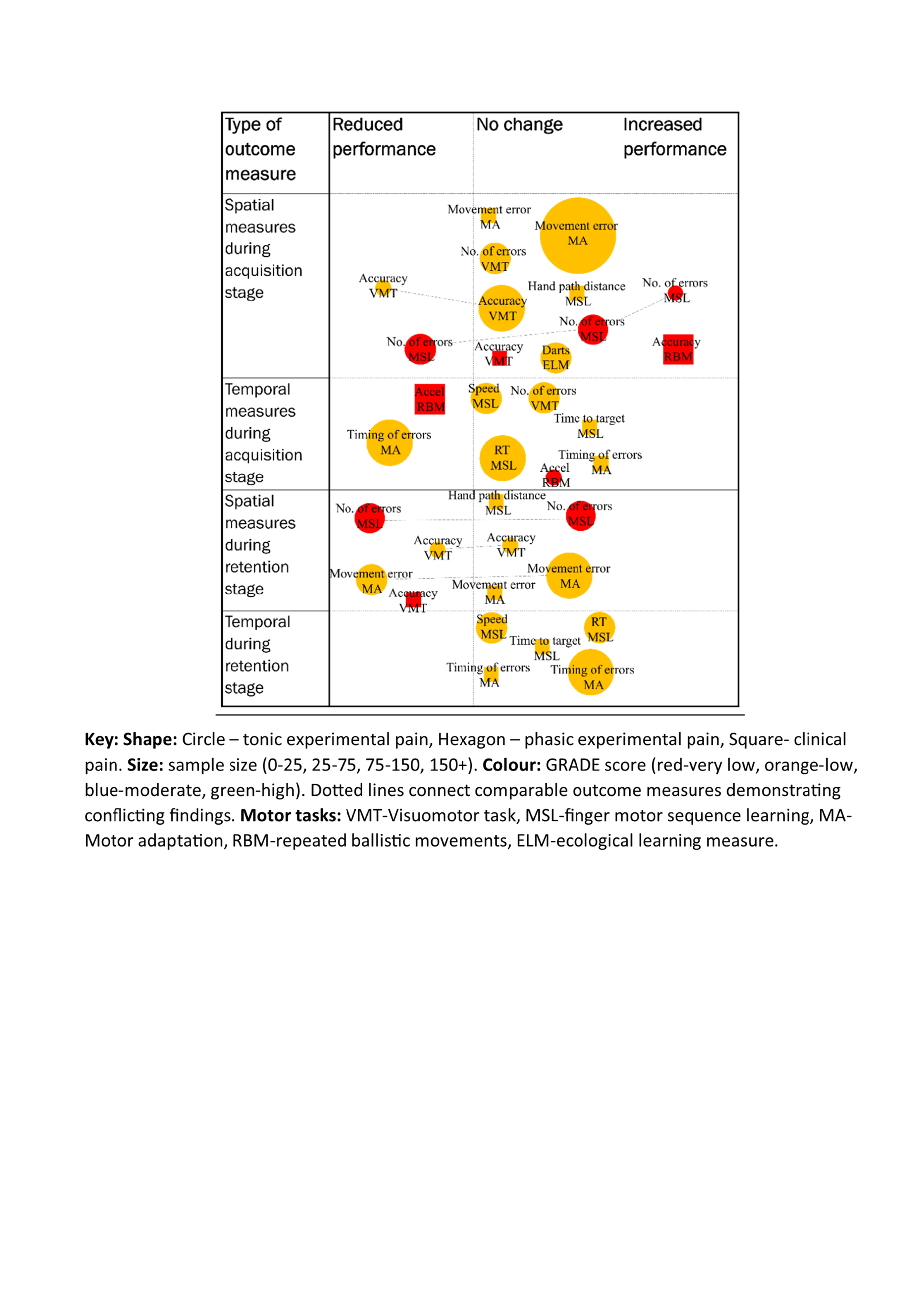

Supplement: S1 Fig — Outcome measures from 18 studies: 15 tonic experimental and five clinical pain. (TIF) [file pone.0274403.s001.tif]

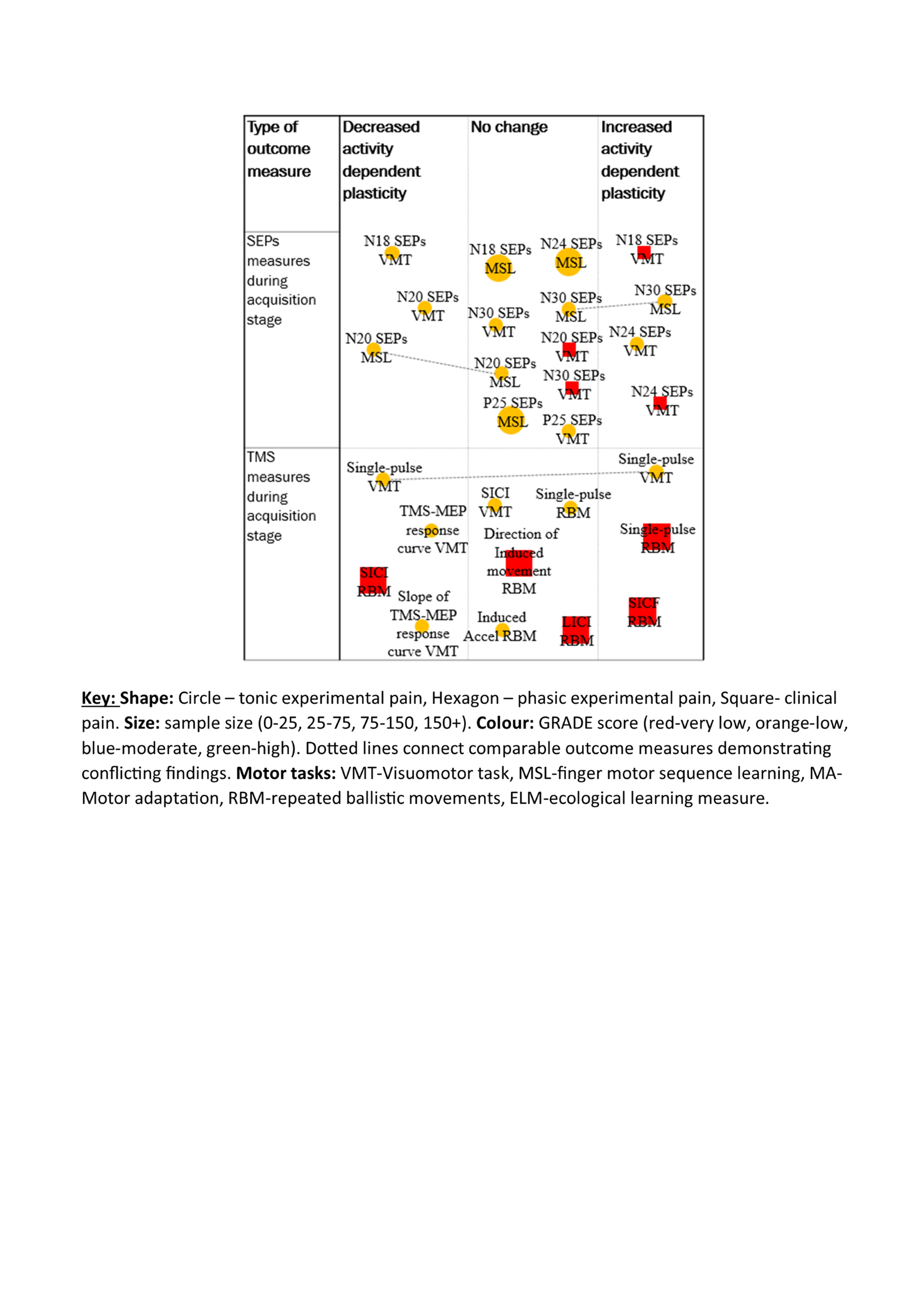

Supplement: S2 Fig — Outcome measures from 18 studies: 15 tonic experimental and five clinical pain. (TIF) [file pone.0274403.s002.tif]
