# Supplementary material for: Exploring pain interference with motor skill learning in humans: A systematic review
Source: PLoS One. 2022 Sep 13;17(9):e0274403. doi: 10.1371/journal.pone.0274403 (PMC9470002; doi:10.1371/journal.pone.0274403)
Supplement: S1 Table — (DOCX) [file pone.0274403.s003.docx]

| Table 8  Summary of GRADE judgements for individual task performance measures for tonic pain. | | | | | | | | |
| --- | --- | --- | --- | --- | --- | --- | --- | --- |
| Outcome Measure | **Studies** | **Motor learning paradigm** | **Indirectness** | **Imprecision** | **Inconsistency** | **Publication bias** | **Overall Risk of bias** | **Quality of evidence** |
| Task performance measures during acquisition | | | | | | | | |
| Accuracy – number of errors. | Bilodeau (2016) [31]  Dancey (2014) [30]  Dancey (2016) [32] | Sequential finger tapping task | Not serious | Serious | Not serious | Not suspected | High  High  High | Very low |
| Accuracy – temporal/spatial error from actual trace | Boudreau (2007) [10]  Dancey (2016) [33]  Dancey (2019) [16]  Rittig-Rasmussen (2014) [35] | Visuomotor task | Not serious | Serious | Not serious | Not suspected | Some concerns  Some concerns  Some concerns  Some concerns | Low |
| Number of errors - missed targets | Mavromatis (2017) [34] | Visuomotor task | Not serious | Serious | Not serious | Not suspected | Some concerns | Low |
| Ecological measure | Arieh (2021) [41] | Dart throwing | Not serious | Serious | Not serious | Not suspected | Some concerns | Low |
| Movement error | Bouffard (2014) [39]  Bouffard (2016) [38]  Lamothe (2014) [36]  Bouffard (2018) [40]  Salomoni (2019) [37] | Motor adaptation task | Not serious | Serious | Not serious | Not suspected | Some concerns Some concerns  Some concerns  Some concerns  Some concerns | Low |
| Speed | Bilodeau (2016) [31] | Sequential finger tapping task | Not serious | Serious | Not serious | Not suspected | Some concerns | Low |
| Movement time | Mavromatis (2017) [34] | Visuomotor task | Not serious | Serious | Not serious | Not suspected | Some concerns | Low |
| Reaction times | Dancey (2014) [30]  Dancey (2016) [32] | Sequential finger tapping task | Not serious | Serious | Not serious | Not suspected | Some concerns  Some concerns | Low |
| Accuracy/speed trade off | Mavromatis (2017) [34] | Visuomotor task | Not serious | Serious | Not serious | Not suspected | Some concerns | Low |
| Timing of errors | Bouffard (2016) [38]  Bouffard (2018) [40] | Motor adaptation task | Not serious | Serious | Not serious | Not suspected | Some concerns  Some concerns | Low |
| Acceleration | Ingham (2011) [13] | Repeated ballistic movements | Not serious | Serious | Not serious | Not suspected | Some concerns | Low |
| Task performance measures during retention | | | | | | | | |
| Accuracy – number of errors. | Bilodeau (2016) [31]  Dancey (2016) [32] | Sequential finger tapping task | Not serious | Serious | Not serious | Not suspected | High  High | Very low |
| Accuracy – temporal/spatial error from actual trace | Dancey (2016) [33]  Dancey (2019) [16] | Visuomotor task | Not serious | Serious | Not serious | Not suspected | Some concerns | Low |
| Movement error | Bouffard (2014) [39]  Bouffard (2016) [38]  Lamothe (2014) [36]  Bouffard (2018) [40]  Salomoni (2019) [37] | Motor adaptation task | Not serious | Serious | Not serious | Not suspected | Some concerns  Some concerns  Some concerns  Some concerns  Some concerns | Low |
| Speed | Bilodeau (2016) [31] | Sequential finger tapping task | Not serious | Serious | Not serious | Not suspected | Some concerns | Low |
| Reaction times | Dancey (2016) [32] | Sequential finger tapping task | Not serious | Serious | Not serious | Not suspected | Some concerns | Low |
| Relative timing of errors | Bouffard (2016) [38]  Bouffard (2018) [40] | Motor adaptation task | Not serious | Serious | Not serious | Not suspected | Some concerns  Some concerns | Low |

| Table 9  Summary of GRADE judgement for individual activity-dependent plasticity measures for tonic pain. | | | | | | | | |
| --- | --- | --- | --- | --- | --- | --- | --- | --- |
| Outcome Measure | **Studies** | **Motor learning paradigm** | **Indirectness** | **Imprecision** | **Inconsistency** | **Publication bias** | **Overall Risk of bias** | **Quality of evidence** |
| Activity-dependent plasticity measure during acquisition – somatosensory evoked potentials | | | | | | | | |
| *SEP Peaks* | Dancey (2016) [33]  Dancey (2014) [30]  Dancey (2016) [32] | Visuomotor Task  Sequential finger tapping task | Not serious | Serious | Not serious | Not suspected | Some concerns  Some concerns  Some concerns | Low |
| Activity dependent plasticity measure during acquisition - TMS measures | | | | | | | | |
| *Single pulse MEPs* | Boudreau (2007) [10]  Mavromatis (2017) [34]  Ingham (2011) [13] | Visuomotor Task  Repeated ballistic movements | Not serious | Serious | Serious | Not suspected | Some concerns  Some concerns  Some concerns | Low |
| SICI | Mavromatis (2017) [34] | Visuomotor Task | Not serious | Serious | Not serious | Not suspected | Some concerns | Low |
| TMS-MEP response curves | Boudreau (2007) [10] | Visuomotor Task | Not serious | Serious | Not serious | Not suspected | Some concerns | Low |
| Slope of TMS-MEP response curves | Dancey (2019) [16] | Visuomotor Task | Not serious | Serious | Not serious | Not suspected | Some concerns | Low |
| Abbreviations: SEP = Somatosensory evoked potential, TMS = Transcranial magnetic stimulation, MEP = Motor evoked potential, SICI = short intracortical inhibition. | | | | | | | | |

| Table 10  Summary of GRADE judgement for individual activity-dependent plasticity and task performance measures for clinical pain. | | | | | | | | |
| --- | --- | --- | --- | --- | --- | --- | --- | --- |
| Outcome Measure | **Studies** | **Motor learning paradigm** | **Indirectness** | **Imprecision** | **Inconsistency** | **Publication bias** | **Overall Risk of bias** | **Quality of evidence** |
| Task performance measures during acquisition | | | | | | | | |
| Accuracy – temporal/spatial error from actual trace | Andrew (2018) [46] | Visuomotor task | Not serious | Serious | Not serious | Not suspected | Serious | Very low |
| Accuracy – direction of voluntary twitches | Parker (2017) [42] | Repeated ballistic movements | Not serious | Serious | Not serious | Not suspected | Serious | Very low |
| Acceleration | Vallence (2013) [43] | Repeated ballistic movements | Not serious | Serious | Not serious | Not suspected | Serious | Very low |
| Time to target | Brown (2022) [44] | Motor sequence task | Not serious | Serious | Not serious | Not suspected | Moderate | Low |
| Hand path distance | Brown (2022) [44] | Motor sequence task | Not serious | Serious | Not serious | Not suspected | Moderate | Low |
| Movement error | Dupuis (2022) [45] | Motor adaptation | Not serious | Serious | Not serious | Not suspected | Moderate | Low |
| Timing of errors | Dupuis (2022) [45] | Motor adaptation | Not serious | Serious | Not serious | Not suspected | Moderate | Low |
|  |  |  |  |  |  |  |  |  |
| Task performance measures during retention | | | | | | | | |
| Accuracy – temporal/spatial error from actual trace | Andrew (2018) [46] | Visuomotor task | Not serious | Serious | Not serious | Not suspected | Serious | Very Low |
| Time to target | Brown (2022) [44] | Motor sequence task | Not serious | Serious | Not serious | Not suspected | Moderate | Low |
| Hand path distance | Brown (2022) [44] | Motor sequence task | Not serious | Serious | Not serious | Not suspected | Moderate | Low |
| Movement error | Dupuis (2022) [45] | Motor adaptation | Not serious | Serious | Not serious | Not suspected | Moderate | Low |
| Timing of errors | Dupuis (2022) [45] | Motor adaptation | Not serious | Serious | Not serious | Not suspected | Moderate | Low |
| Activity dependent plasticity measure during acquisition – somatosensory evoked potentials | | | | | | | | |
| SEP Peaks | Andrew (2018) [46] | Visuomotor Task | Not serious | Serious | Not serious | Not suspected | Serious | Very low |
| Activity dependent plasticity measure during acquisition - TMS measures | | | | | | | | |
| Single pulse MEPs | Vallence (2013) [43] | Repeated ballistic movements | Not serious | Serious | Not serious | Not suspected | Serious | Very low |
| SICI | Parker (2017) [42] | Repeated ballistic movements | Not serious | Serious | Not serious | Not suspected | Serious | Very low |
| SICF | Parker (2017) [42] | Repeated ballistic movements | Not serious | Serious | Not serious | Not suspected | Serious | Very low |
| LICI | Parker (2017) [42] | Repeated ballistic movements | Not serious | Serious | Not serious | Not suspected | Serious | Very low |
| Direction TMS induced twitches | Parker (2017) [42] | Repeated ballistic movements | Not serious | Serious | Not serious | Not suspected | Serious | Very low |
| Abbreviations: SEP = Somatosensory evoked potential, TMS = Transcranial magnetic stimulation, MEP = Motor evoked potential, SICI = short intracortical inhibition, SICF = short intracortical facilitation, LICI = Long intracortical inhibition. | | | | | | | | |
