# Supplementary material for: Exploring pain interference with motor skill learning in humans: A systematic review
Source: PLoS One. 2022 Sep 13;17(9):e0274403. doi: 10.1371/journal.pone.0274403 (PMC9470002; doi:10.1371/journal.pone.0274403)
Supplement: S1 File — (PDF) [file pone.0274403.s004.pdf]

## **Search Strategy**

Example of MEDLINE search;

( ( TITLE-ABS-KEY ( ( ( pain OR nociception OR noxious OR *algia OR athralgia OR myalgia OR neuralgia OR *dynia ) W/10 ( interfere* OR *ffect* OR impair* OR impact* OR associat* OR imped* OR change* OR alter* OR disturb* OR influence* OR modif* OR determine* OR reduc* ) ) ) ) AND ( TITLE-ABS-KEY ( ( performance OR *plasticity OR *excitability ) W/10 ( training OR learning OR acquisition OR practice ) ) ) AND ( TITLE-ABS-KEY ( ( performance OR *plasticity OR *excitability ) W/10 ( *motor OR skill OR task ) ) ) ) OR ( ( TITLE-ABS-KEY ( ( ( pain OR nociception OR noxious OR *algia OR athralgia OR myalgia OR neuralgia OR *dynia ) W/10 ( interfere* OR *ffect* OR impair* OR impact* OR associat* OR imped* OR change* OR alter* OR disturb* OR influence* OR modif* OR determine* OR reduc* ) ) ) ) AND ( ( TITLE-ABS-KEY ( "*motor training" OR "*motor learning" OR "*motor acquisition" OR "*motor practice" OR "Skill training" OR "Skill learning" OR "Skill acquisition" OR "Skill practice" OR "Task learning" OR "task training" OR "Task acquisition" OR "Task practice" ) ) OR ( TITLE-ABS-KEY ( "*motor adaptation" OR "motor sequence learning" OR "repeated practice" ) ) ) )
