# Supplementary material for: Exploring pain interference with motor skill learning in humans: A systematic review
Source: PLoS One. 2022 Sep 13;17(9):e0274403. doi: 10.1371/journal.pone.0274403 (PMC9470002; doi:10.1371/journal.pone.0274403)
Supplement: Attachment — Submitted filename: Response to Reviewers.docx [file pone.0274403.s006.docx]

**Please see below table outlining detailed response to each point made by the reviewer.**

| Reviewer's comments | Response |
| --- | --- |
| 1. Please ensure that your manuscript meets PLOS ONE's style requirements, including those for file naming. | **Authors response: Changes made to title page and formatting of titles. File naming uploaded as per guidelines on PLOSone website.** |
| 2. Please include your tables as part of your main manuscript and remove the individual files. Please note that supplementary tables (should remain/ be uploaded) as separate "supporting information" files. | **Authors response: All tables uploaded to manuscript. Supplementary figures and tables uploaded separately. Titles of supplementary material added to bottom of manuscript.** |
| 3. How does the paper differ from recent work by Izadi et al., [1] examining motor learning in response to experimental pain models? Be sure to compare and contrast findings in discussion. Be sure to discuss how findings compare and contrast with recent reviews [1,2] and findings in clinical pain [3]. Discuss limitations of current approach and how current findings compare with recent work. | **Authors response: Two systematic reviews (Izadi et al. (2022) and Stanisic et al. (2022) have been published in this research area since the original submission of this manuscript. The below comments have been added to the introduction and discussion to reflect this and discuss the different methodological approaches and review findings. A discussion around the impacts of pain on cortical excitability has been adapted. The findings of the article mentioned by the reviewer [2] exploring the impact of chronic pain on cortical excitability (Parker et al 2016) has been considered when interpretating the results of this review. A section on limitations has been added to the discussion.**  **Lines 78-86 added to intro:**  *‘Two systematic reviews [36,68] have been recently published in this area. Izadi et al. [36] investigated the impact of acute experimental pain on behavioural performance following motor learning. The authors concluded that the majority of studies reported negative impacts of acute pain on motor learning. Results were grouped based on the tissue where the pain was induced and investigated only experimental tonic pain paradigms. Stanisic et al. [68] explored the impact of experimental and clinical pain on motor learning induced neuroplasticity, assessed using TMS. They reported both acute and clinical pain may impede neuroplasticity, resulting from motor learning, while highlighting a diversity of study findings. Neither of the above systematic reviews differentiated the impact of different motor learning paradigms on this interaction despite identifying that this may influence the findings.’*  ***Lines 90-94 added to intro:***  *‘This is the first systematic review to delineate the impact of experimental and clinical pain on a multitude of different behavioral and neuroplasticity outcomes measures and look to compare and contrast these findings related to different pain paradigms (experimental tonic or phasic pain and clinical pain) and different motor paradigms (motor sequence learning, visuomotor learning, repeated ballistic movements, motor adaptation and ecological learning).’*  **Lines 514-517 added to discussion:**  *‘The findings in this review are consistent with a systematic review by Stanisic et al. [68] who reported acute experimental pain may influence training induced neuroplasticity defined by TMS. The authors suggested the impact of pain on training induced neuroplasticity may be dependent on the motor learning paradigms utilised.’*  **Lines 551-576 adapted in discussion to considering findings Parker et al 2016 [53]:**  *‘Interference of motor learning has been shown to occur when two motor tasks compete for the same neural substrate [14,66]. Lange et al. [41] points to inhibition of shared networks as a mechanism underlying interference with motor learning. It has been well documented that pain and movement activate similar areas of the central nervous system including spinal cord, cerebellum, basal ganglia, anterior cingulate cortex, premotor and primary motor cortex [2,19,49,74]. Previous systematic reviews provide evidence that experimental pain decreases excitability of the motor pathway including the motor cortex [6,59,63]. These findings contrast with the findings of this study which found no consistent evidence that experimental pain inhibits cortical changes associated with motor learning . Sub-grouping analysis performed by Sanderson et al. [63] suggests that the impact of pain on cortical excitability may be dependent on the specific pain paradigm or tissue stimulated. The authors reported increased cortical excitability when hypertonic saline was injected into non-contractile tissue and a decrease when injected into contractile tissue. Only four studies out of the 15 experimental pain studies included in the present review induced pain in muscle tissue and therefore further research is needed to explore whether the physiological effects on motor learning are different from cutaneous induced pain models.*  *In agreement with this review, Parker et al. [53] and Sanderson et al. [63] report no consistent evidence that clinical pain alters motor cortical excitability measured through single pulse measures. In contrast, Parker et al. [53] reported a reduction in intra-cortical inhibition, a mechanism linked to GABAergic inhibition and potential modulator of motor learning, in chronic pain populations. The majority of studies included in this review utilised single pulse measures of cortical excitability and therefore it is not possible to come to any conclusions around the impact of clinical pain on disinhibition of cortical pathways following motor learning. It is possible that pain interference with motor learning may be specific to pain paradigms and/or sub-groups of a population that demonstrate disruption of the normal inhibitory neural networks within the motor pathways, such as neuropathic pain [53]. Further discussions regarding this are limited by the fact that both clinical pain populations employed by the two studies included in this review had an absence in symptoms associated with the above neural changes, assessed using pain pressure thresholds and sensory perception.’*  **Lines 585-601 added to discussion:**  *5. Strengths and limitations*  *This is the first systematic review to provide an in-depth analysis of the impacts of both experimental pain and clinical pain on individual outcome measures for different motor learning paradigms. Employing this approach has attempted to minimise comparisons between motor learning paradigms which potentially have different underlying neural mechanisms [24], and outcome measures which measure efficiency and effectiveness versus those which explore performance strategies. Consequently, this review demonstrates, across different motor learning paradigms, efficiency and effectiveness of performance can significantly increase across learning despite the presence of pain and alternative motor strategies. This is contrary to the conclusions reported by a recent systematic review [36] but likely reflects the different approach outlined above. The limitations of this review need to be considered when interpreting the findings. Due to the heterogeneity of the motor learning paradigms, pain paradigms and outcome measures a meta-analysis was not used. Overall, there was limited confidence in the impact of pain on most outcome measures due in part to small sample sizes and the assigned level of risk of bias for each study. In an attempt to reduce heterogeneity of experimental design and competing physiological mechanisms, studies using interventions to lessen pain prior to motor learning and prism-adaptation paradigms were excluded. It is possible that omitting these studies may have rejected findings that may have contributed different perspectives to the research question.* |
| 4. Why were activity dependent plasticity measures limited to amplitude, temporal, or spatial characteristics? Why were frequency domain measures not considered? | **Authors response: Activity measures were not limited to amplitude, temporal or spatial measures and the method has been adjusted to reflect this. No frequency domains of SEPs were explored as a measure in any included study and therefore no further discussion on this was deemed appropriate. To clarify this a sentence was added to the results.**  **Line 128 adapted as follows:**  *‘Activity dependent plasticity measures included, but not limited to, changes in amplitude, temporal or spatial characteristic obtained using magnetic resonance imaging (MRI), transcranial magnetic stimulation (TMS) or electroencephalogram (EEG).’*  **Lines 236-238 added:**  *‘The four studies exploring SEPs collected data on the amplitude (mV) of the early SEP peaks only.’* |
| 5. Why were studies including treatments as an adjunct to motor learning excluded? Wouldn’t those studies provide significant utility in evaluating potential mechanisms by which learning could be improved in the pretense of pain? | **Authors response: Scoping searches prior to the preparation of the systematic review protocol revealed two studies including other treatments alongside motor learning tasks. These included manipulation (Baarbe et al., 2018) and repeated peripheral magnetic neurostimulation (Massie-Alarie., 2017). The authors agreed including these articles would add to the existing diversity of the reviews methods which would impact on the ability for the review to provide coherent summary of research in this area for readers.**  **Lines 133-136 adapted to:**  *‘Also excluded were any study including treatments as an adjunct to motor learning, in an attempt to minimise heterogeneity of interventions subjects were exposed to and consequently minimise confounding variables that may impact on the amount of learning observed’* |
| 6. The search should be updated to reflect new papers published in the past year. | **Authors response: Search updated to July 2022, see line 138. Two further studies found meeting the criteria and included in the analysis. Two systematic reviews exploring similar research questions were found and these were included in the intro and discussion (see point 3).**  **Information on two addition studies added to results and discussion – see lines 395-432 & 536-549.** |
| 7. Line 81-84: In my opinion, a brief introduction to the three pain paradigms and various motor paradigms might be needed to enable the readers to understand the differences. | **Authors response: Definitions of pain paradigms added to text and motor learning definitions included in new table (Table 1). Line 118.**  **Added in method line 107-111.**  *‘Studies were eligible for inclusion in this review if they included an experimental group consisting of adults (age ≥ 18 years old) who experienced either experimental pain (tonic (long lasting) or phasic (brief noxious stimuli)) or clinical pain. Clinical pain was considered as any symptom of pain included in the IASP definition for pain [56] excluding those occurring in the presence of neurological disease or due to delayed onset of muscle soreness.’* |
| 8. Line 404-406: I recommend the authors elaborate more on how somatosensory feedback affects task performance. | **Authors response: Further research exploring the impact of somatosensory feedback/processing is added to the discussion including changes at different levels of the sensory pathway.**  **Lines 468-478 added in discussion:**  *‘Researchers have theorised that somatosensory feedback plays a vital role in skill acquisition. Evidence from animal studies have demonstrated impaired learning of novel movements in the presence of lesions of the primary somatosensory cortex [55]. Similar findings have been demonstrated in humans using a repetitive TMS virtual somatosensory cortex lesion model [73]. In addition, peripheral manipulation of sensory information using lidocaine has been shown to decrease overall performance, increase errors and reduce reaction times during the same tongue protrusion task used by Boudreau and colleagues [10]. Both Vidoni et al. [73] and Boudreau et al. [10] utilised complex tasks requiring both visual information and graded movements during performance. Caution should be taken when comparing research using central and peripheral somatosensory disruptions, as mechanisms underlying central and peripheral sensorimotor interactions may differ [73].’* |
| 9. In my opinion, the experimental pain (especially the way to apply capsaicin) would not really damage the human tissue but other ways might cause some injuries, which might result in different physiological changes. The evident difference should be noted and discussed. | **Authors response: Capsaicin models were included in this study and were not considered to be a paradigm that may result in tissue damage. The method outlines those pain paradigms that were excluded due to be associated with potential changes in the muscle tissue such as DOMS (related to micro tears in the muscle and a healing process) and neurological disease (associated with reduced neural drive and associated muscle wasting). The authors agree clinical pain studies can be associated with tissue damage. This is highlighted during discussions around ROBIN-I, risk of bias and future studies. Musculoskeletal/neurological changes associated with the pain conditions could act as confounding variables. A lack of screening of clinical pain populations at baseline for the above confounding variables was a key determinant of the serious risk of bias judgements and subsequent exclusion of those studies from further analysis. The remaining two clinical pain studies included for further analysis did not differ in terms of baseline measures related to motor and sensory testing and therefore further discussions on this was not included.**  **Lines 110-111 outlines exclusion of conditions causing muscle damage:**  *‘excluding those occurring in the presence of neurological disease or due to delayed onset of muscle soreness.’*  **Lines 254-259 adapted in results discussing screening for muscle damage of clinical studies:**  *‘Three out of the five clinical studies assessed using ROBIN-I were classified as ‘serious’. The ‘serious’ classifications were for the ‘confounding variables’ section, related to not controlling or capturing information on potential sensory and motor deficits, associated with the clinical population, that may directly influence engagement in the task. Measures of the amount of pain participants were experiencing during the motor learning sessions was also lacking in these studies. The other two clinical studies were judged to have a ‘moderate’ risk of bias.‘*  **Lines 555-564 added to suggest impact of pain on cortical excitability may depend on physiological changes associated with experimental pain paradigms.**  *‘Previous systematic reviews provide evidence that experimental pain decreases excitability of the motor pathway including the motor cortex [6,59,63]. These findings contrast with the findings of this study which found no consistent evidence that experimental pain inhibits cortical changes associated with motor learning . Sub-grouping analysis performed by Sanderson et al. [63] suggests that the impact of pain on cortical excitability may be dependent on the specific pain paradigm or tissue stimulated. The authors reported increased cortical excitability when hypertonic saline was injected into non-contractile tissue and a decrease when injected into contractile tissue. Only four studies out of the 15 experimental pain studies included in the present review induced pain in muscle tissue and therefore further research is needed to explore whether the physiological effects on motor learning are different from cutaneous induced pain models.’* |
| 10. Line 415-416: It is interesting that the increased attention of participants caused by pain might facilitate task performance. Is that possible that other sensory inputs would cause similar results? | **Authors response: The following paragraph was adapted for clarity, the research referred to when exploring impact of attention on neuroplastic changes used non-noxious sensory stimuli or auditory commands. A further question posed at the end of this paragraph is whether the attentional changes are a result of the painful stimulation or any sensory information and the importance of choice of control groups to explore this.**  **Lines 489-494 adapted:**  *‘Previous research, utilising other sensory inputs such as non-noxious electrical stimuli to the limb completing the learning task, reported increased attention facilitates neuroplasticity [61], measured using TMS. Whereas impairment in behavioural performance following motor learning occurs when instructions are given to intentionally divert attention away [69]. The latter is consistent with findings by Ingham et al. [35]. The authors reported decreased training induced changes in excitability related to hand motor learning in the presence of experimentally induced knee pain.‘* |
| 11. In the clinical pain studies, were they all acute pain or chronic pain? Acute pain (e.g., acute ankle sprain) and chronic pain (e.g., osteoarthritis) might influence motor learning a lot. Much evidence has pointed out that chronic pain would change the excitability level or activation area of the cerebral cortex. I recommend the authors could put the related findings in the discussion. | **Authors response: The two included clinical studies after quality assessment were both chronic pain paradigms and this has been added to the discussion. The studies found no effect of chronic pain on learning and therefore does not suggest differences between chronic and experimental acute pain effects on learning. A caveat to this, stated in the discussion, is sub-groups of chronic pain patients such as neuropathic pain may have different interactions with learning and chronic pain and acute pain mechanisms differ. The 3 clinical pain studies removed from the narrative review all explored different types of chronic pain with only one study demonstrating impact on motor learning. As mentioned previously as sensory and motor symptoms were not collated in these studies it is difficult to subgroup these studies. Worth noting one of these studies looked at osteoarthritis of the thumb and found no change in behaviour learning outcomes resulting from motor learning. In response to the comment stating chronic pain changes excitability of the cortex, two systematic reviews (including Parker et al 2016) are presented in the discussion which conclude that there is evidence of no impact of chronic pain on single pulse measures of cortical excitability but possibly changes in intracortical inhibition.**  ***Lines 535-543 added discussing pain paradigms in clinical studies.***  *‘The two clinical pain studies [15,27] included in this review investigated the impact of clinical pain on task performance measures only. Both studies included participants with chronic pain only, defined as those experiencing symptoms for more than 3 months. No impact of clinical pain was reported on the four outcome measures during either acquisition or retention. This provides limited evidence that chronic pain effects on motor learning are similar to acute experimental pain effects discussed above. Although, caution must be taken when comparing the effects of acute and chronic pain. Research has suggested that both the global and local neural activity of chronic clinical pain states differs from acute pain states [3,5,42,64,74].’*  ***Lines 565-576 discusses research on the impact of clinical pain on cortical excitability.***  *‘In agreement with this review, Parker et al. [53] and Sanderson et al. [63] report no consistent evidence that clinical pain alters motor cortical excitability measured through single pulse measures. In contrast, Parker et al. [53] reported a reduction in intra-cortical inhibition, a mechanism linked to GABAergic inhibition and potential modulator of motor learning, in chronic pain populations. The majority of studies included in this review utilised single pulse measures of cortical excitability and therefore it is not possible to come to any conclusions around the impact of clinical pain on disinhibition of cortical pathways following motor learning. It is possible that pain interference with motor learning may be specific to pain paradigms and/or sub-groups of a population that demonstrate disruption of the normal inhibitory neural networks within the motor pathways, such as neuropathic pain [53]. Further discussions regarding this are limited by the fact that both clinical pain populations employed by the two studies included in this review had an absence in symptoms associated with the above neural changes, assessed using pain pressure thresholds and sensory perception.’* |
| 12. Table 1 and Table 2. Please provide the full term for abbreviations in the footnotes like Table 3 | **Authors response: Footnotes/abbreviations added to all tables.** |

[1] Izadi M, Franklin S, Bellafiore M, Franklin DW. Motor Learning in Response to Different Experimental Pain Models Among Healthy Individuals: A Systematic Review. Front Hum Neurosci. 2022;16:863741. Published 2022 Mar 24. doi:10.3389/fnhum.2022.863741

[2] Parker, Rosalind S., et al. "Is motor cortical excitability altered in people with chronic pain? A systematic review and meta-analysis." Brain stimulation 9.4 (2016): 488-500.

[3] Brown, Michael R., Kirkwood E. Personius, and Jeanne Langan. "Participants with mildly-disabling chronic neck pain perform differently during explicit compared to implicit motor learning of a reaching task." PloS one 17.4 (2022): e0266508
